# Comparative Study of Ferrocene- and Indene-Based Tamoxifen Derivatives of Different Molecular Flexibility on High-Mortality Cancer Cell Lines

**DOI:** 10.3390/ph18091417

**Published:** 2025-09-20

**Authors:** Márton Kalabay, Zsófia Szász, Eszter Lajkó, Bálint Bagu, Éva Pállinger, Cintia Duró, Tamás Jernei, Antal Csámpai, Angéla Takács, László Kőhidai

**Affiliations:** 1 Department of Genetics, Cell- and Immunobiology, Faculty of Medicine, Semmelweis University, Nagyvárad Square 4, 1085 Budapest, Hungary; kalabay.marton.kristof@semmelweis.hu (M.K.); szasz.adrienn.zsofia@semmelweis.hu (Z.S.); lajko.eszter@semmelweis.hu (E.L.); bagu.balint@stud.semmelweis.hu (B.B.); pallinger.eva@semmelweis.hu (É.P.); kohlasz2@gmail.com (L.K.); 2 Department of Inorganic Chemistry, Faculty of Chemistry, Eötvös Loránd University, Egyetem Square 1-3, 1053 Budapest, Hungary; cinti1994@gmail.com (C.D.); jernei91@gmail.com (T.J.); antal.csampai@ttk.elte.hu (A.C.)

**Keywords:** tamoxifen, breast cancer, pancreatic cancer, ferrocene, indene, ROS, apoptosis

## Abstract

Tamoxifen is a well-established selective estrogen receptor modulator (SERM) widely used in breast cancer treatment, yet its efficacy varies across tumor types. To enhance its antitumor potential, we previously synthesized and investigated novel ferrocene-linked (T5, T15) derivatives. This publication is a close continuation of this work, introducing a new indene-based (T6) derivative. **Objectives**: The main aim of this study was to further broaden our knowledge of the mechanism behind the increased antitumor effect of the ferrocene-linked drugs (T5 and T15) and compare it with a new, indene-based tamoxifen derivative, T6. The indene moiety was selected as a rigid, hydrophobic aromatic unit to probe pharmacological effects independent of ferrocene’s redox activity. **Methods**: The compounds were tested on MCF7, MDA-MB231 and PANC1 cells. Cell viability was assessed with the AlamarBlue assay and the xCELLigence SP system. Reactive oxygen species (ROS) production was measured with the ROS Glo assay. Flow cytometry and RT-qPCR experiments were conducted to assess apoptosis and ROS regulation as well. **Results**: The modified compounds demonstrated an increased cell-viability-decreasing effect in breast (MCF7, MDA-MB-231) and pancreatic (PANC1) cancer cell lines, influencing both estrogen-receptor-dependent and -independent pathways. T6 led to G2/M phase arrest in PANC1 cells. Beyond cell cycle disruption, these derivatives significantly elevated ROS levels, contributing to apoptosis. **Conclusions**: Our findings suggest that these structural modifications retain tamoxifen’s pharmacophore properties while expanding its mechanism of action, particularly through universal interactions independent of the ER status of tumor cells. The enhanced antitumor effects highlight the potential of these derivatives as promising candidates for improved cancer therapies.

## 1. Introduction

Tamoxifen was initially identified as a contraceptive named ICI 46,474 by Dora Richardson et al. in 1962, and today it stands as the benchmark treatment for estrogen receptor (ER)-positive breast cancer [1]. However, its potential extends beyond oncology, which is evident in its diverse applications. Over the past two decades, it has become evident that tamoxifen not only acts as a selective estrogen receptor modulator (SERM), exhibiting agonistic or antagonistic effects on ER in a tissue-specific manner, but also exerts various off-target effects unrelated to ER. In the contemporary landscape of targeted tumor therapy, tamoxifen has sparked renewed interest, leading to extensive research exploring its multifaceted properties.

The ongoing pursuit aims to identify novel disease indications for tamoxifen or enhance its therapeutic efficacy through molecular modifications. While tamoxifen shows promise across a broad range of diseases, efforts are underway to develop modified derivatives that surpass the parent compound. Ferrocene, or carbo- and heterocycles, can be attached to the pharmacophore base of tamoxifen, augmenting its properties. Ferrocene linkage imparts advantageous redox properties [2], and carbo- and heterocycles serve as platforms for further selective modifications targeting specific ER isoforms [3].

A previous publication by our research group described two tamoxifen derivatives with improved antitumor effects compared to the parent molecule. This research article is a close continuation of our previous experimental series [4].

**Scheme 1:**
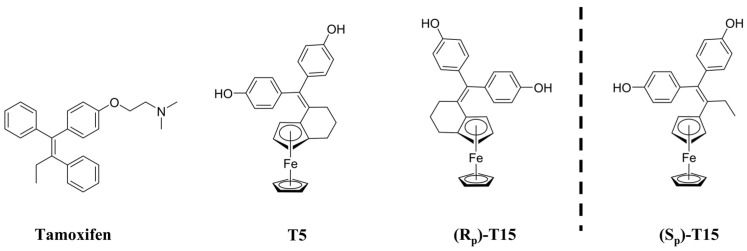
Chemical structure of tamoxifene and the ferrocene-linked derivatives T5 and T15 [4].

To briefly summarize our previous publication, we tested out tamoxifen, T5, and the racemate of the planar chiral T15 (ferrocene-linked derivatives) (Scheme 1) on three cell lines: MCF7 (ER-positive breast adenocarcinoma), MDA-MB231 (ER-negative breast adenocarcinoma), and PANC1 (pancreas adenocarcinoma).

Immunocytochemistry revealed that the cell lines used in our experiment significantly differ in their estrogen receptor expression profile. The three most well-known estrogen receptor isoforms are the ERα, Erβ, and the G-protein coupled estrogen receptor 1 (GPER1). ERα stands out as the predominant nuclear ER isoform, with pathological diagnostics continuing to assess tumor ER positivity primarily based on ERα presence. In contrast, ERβ, another nuclear ER isoform, exhibits higher expression in breast and endometrial tissues but holds less significance in their physiological proliferation compared to ERα. Despite abundant data on ERβ expression, its exact role in breast cancer development and progression remains unclear. Our findings indicate that all three cell lines express ERα, whereas only MCF7 and MDA-MB-231 express ERβ. G-protein-coupled Estrogen Receptor 1 (GPER1) represents a distinct estrogen receptor isoform, functioning as a Gs-coupled seven-transmembrane receptor. However, its cellular localization remains debated, with conflicting evidence suggesting either plasma membrane or endoplasmic reticulum localization [5]. Our previous results suggest predominant localization of GPER1 in the endoplasmic reticulum rather than the plasma membrane. Notably, GPER1 expression levels were highest in MCF7 cells, followed by PANC1 and MDA-MB-231, consistent with previous findings by other researchers [6].

The introduction of the ferrocene component enhances the cell viability, decreasing the impact of tamoxifen on both breast and pancreatic cancer cell lines. The drugs of this study exhibit the ability to halt the cell cycle, albeit in a concentration-dependent manner. This means that cell cycle arrest prevails at concentrations below the compound’s IC_50_ value. Tamoxifen treatment led to S and G1 phase arrest in MCF7 and MDA-MB231 cells, respectively. The ferrocene-linked derivatives arrested the cell cycle on these cell lines in the G1 phase while being cytotoxic on PANC1 pancreatic adenocarcinoma cells.

Further investigation has unveiled several potential mechanisms contributing to the observed effects of the ferrocene-linked tamoxifen derivatives. The primary pathway they engage in depends on the tumor’s estrogen expression profile. In MCF7 cells, where all three isoforms of ER are present, the antagonistic effect on ER-α predominates. However, the presence of GPER1 inhibits the full effectiveness of tamoxifen by upregulating several tumor-protective proteins. Conversely, in MDA-MB-231 cells, where only ERβ is notably expressed, tamoxifen’s antagonistic effect on this isoform is clearly prominent. In PANC1 cells, only GPER1 expression is detectable. PANC1 was selected as a representative aggressive and ER-independent model. While the observed antitumor effects induced by tamoxifen can be attributed to non-estrogen-receptor-dependent mechanisms, the tumor-protective pathways induced by GPER1 interfere with these off-target effects. The ferrocene-linked novel tamoxifen derivatives, T5 and T15, can mitigate the tumor-promoting effects induced by GPER1 through direct viability, decreasing the effect resulting from oxidative stress [4].

**Scheme 2:**
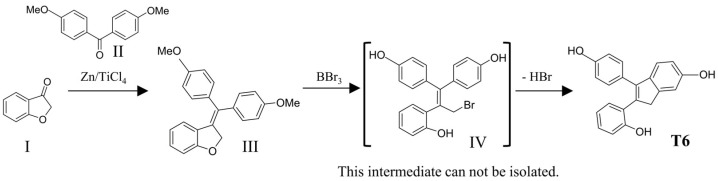
Synthesis of indene-based tamoxifene derivative T6 [7].

Differences in the rotational freedom of the molecules may also be a determining factor in the varying antitumor effects of ferrocene-containing derivatives observed on breast and pancreatic tumor cell lines. Due to this rotational flexibility, these compounds may bind differently to the various ER receptor isoforms expressed in our different tumor cell lines. In the present study, we also investigated another tamoxifen derivative with a different structure in terms of rotational freedom, namely T6 (Scheme 2). Synthesis of T6 has been reported earlier [7].

**Scheme 3:**
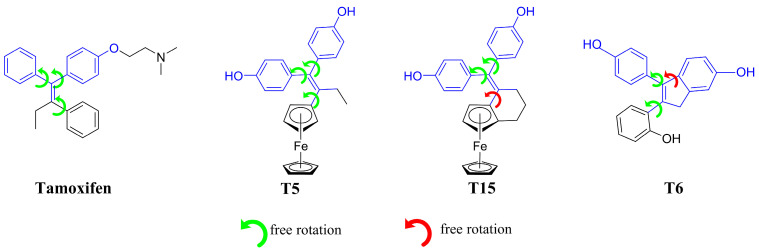
Representation of the different conformational flexibility of the investigated compounds, focusing on whether the bonds of which rotation is free or hindered in the molecular architecture.

As shown in Scheme 3, in terms of rotational freedom in the molecular residue present in all hydroxytamoxifene analogues, the three investigated compounds can be characterized by different conformational flexibility, which may influence their pharmacodynamics. The indene moiety is not expected to contribute to redox activity but may instead modulate lipophilicity and steric orientation of the tamoxifen scaffold [8,9]. This substitution therefore enables investigation of whether improved receptor binding or altered membrane permeability can be achieved through purely organic aromatic modification, independent of the unique redox chemistry of ferrocene [10].

The main aim of this study was to further broaden our knowledge of the molecular mechanism behind the increased antitumor effect of the ferrocene-linked drugs (T5 and T15) and compare it with a new, indene-based tamoxifen derivative, T6 (Scheme 2), on different cancer cell lines, modeling high-mortality tumor types.

## 2. Results

### 2.1. Cell Viability, Cell Cycle Analysis and ROS Production After T6 Treatment

Before comparing the molecular mechanisms of action of the different tamoxifen derivatives, the effects of T6 on cell viability, cell cycle progression, and ROS production were investigated in the tested tumor cell lines and compared to our previously published results for the ferrocene derivatives and the parent compound.

In the case of T6, the prevention of rotation inside the bis-(4-hydroxyphenyl) fragment showed a cell line-specific preference in enhancing a cell viability decrease compared to the parent molecule. While the indene-based T6 molecule resulted in a worse IC_50_ value than its unmodified or ferrocene-linked counterpart in MDA-MB-231 and PANC1 cells, it led to a tenfold improvement in antitumor effect on the classical ER-positive MCF7 breast cancer cell line at 72 h (IC_50_: 4.9 µM—see Table 1). Results measured on Normal Human Dermal Fibroblast (NHDF) cells were added for comparison and to bring an insight into the selectivity index of the derivatives. We highlight that T15 was basically ineffective against the non-malignant cell line, suggesting its safety.

**Figure 1:**
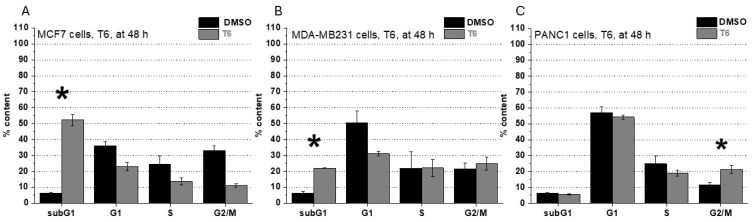
Impact of T6 on the cell cycle at 48 h in MCF7 (**A**), MDA-MB-231 (**B**) and PANC1 cells (**C**). Each cell line was treated with 25 µM. Each result represents the measurement of two parallel samples (*n* = 2). Data are given as mean values  ±  standard deviation (SD). Asterisks mark the subpopulation, where the number of cells is significantly higher compared to the DMSO control, and a cell cycle arrest is seen (*p* < 0.05: *), determined by the one-way ANOVA test followed by Fisher’s LSD post hoc test.

Regarding how it affects the cell cycle progression, T6 showed no significant effect on PANC1 cells in 24 h (Figure S1); however, it elicited a G2/M phase arrest in 48 h (Figure 1C). On MCF7 cells, it did not affect the cell cycle, as it led to the accumulation of cell debris (determined as the subG1 population), but only at a later observed period of time (Figure 1A), while on the other hand, on the MDA-MB231 cell, this effect was observed right after treatment (Figure 1B, Figures S1 and S2).

**Figure 2:**
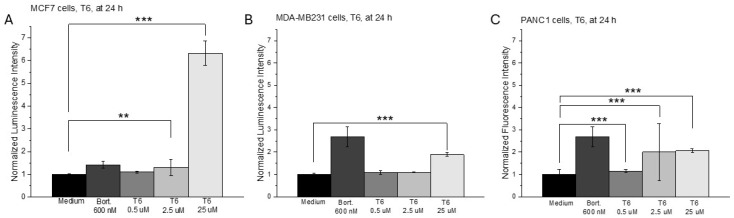
Impact of T6 on the ROS production of the model cells ((**A**): MCF7, (**B**): MDA-MB-231, (**C**): PANC1). Each cell line was treated with 0.5, 2.5 and 25 µM tamoxifen derivatives and 600 nM bortezomib (Bort.) was chosen as a positive control due to its well-known oxidative-stress-increasing property [11]. Each column on the graph represents the measurement of three parallel samples (*n* = 3). Data are given as mean values  ±  standard deviation (SD). Asterisks mark the concentrations, where the ROS production was significantly higher compared to the untreated, negative, medium control (*p* < 0.01: **, *p* < 0.001: ***) determined by the one-way ANOVA test followed by Fisher’s LSD post hoc test.

T6 significantly elevated ROS production on all three investigated cell lines, but only in the highest measured concentration (Figure 2). Subsequently, the most potent ROS production was observed on the MCF7 cell line (Figure 2A), where the IC_50_ value of T6 was also the lowest.

### 2.2. Apoptosis Induction

**Figure 3:**
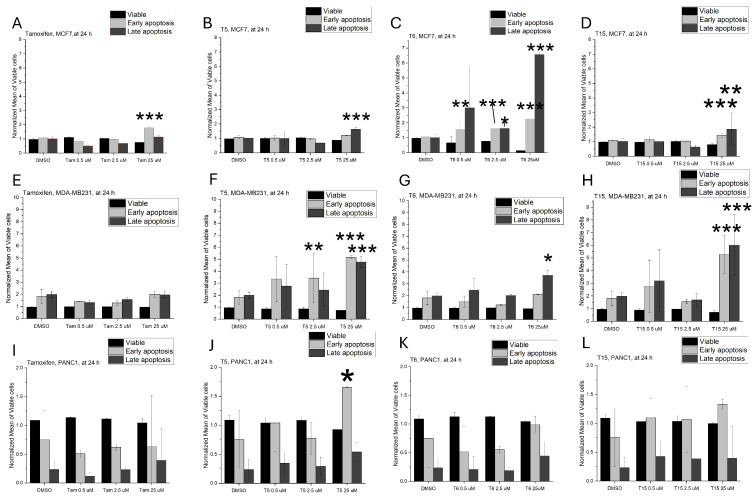
Impact of tamoxifen, T5, T6 and T15 on MCF7 (**A**–**D**), MDA-MB231 (**E**–**H**) and PANC1 (**I**–**L**) cells regarding apoptosis induction. All experiments were performed in triplicate after an incubation period of 24 h. Each cell line was treated with 0.5, 2.5 and 25 µM. Data are given as mean values  ±  standard deviation (SD). Asterisks mark the subpopulation, where the number of cells is significantly higher compared to the DMSO control (*p* < 0.05: *, *p* < 0.01: **, *p* < 0.001: ***) determined by the one-way ANOVA test followed by Fisher’s LSD post hoc test.

On MCF7 cells, tamoxifen induced apoptosis at the highest concentration. Treatment with the ferrocene-linked T5 and T15 derivatives predominantly raised the number of late apoptotic cells. In contrast, the indene-based T6 derivative induced a smaller proportion of early apoptotic cells but led to a more pronounced accumulation of late apoptotic cells (Figure 3A–D and Figure S3).

On MDA-MB231 cells, tamoxifen had no significant effect on apoptotic cell death. The ferrocene-linked derivatives led to significantly higher amounts of both early and late apoptotic cells. Similarly, T6 treatment resulted in an elevated proportion of late apoptotic cells (Figure 3E–H and Figure S4).

On PANC1 cells, only the ferrocene-linked T5 induced apoptosis, with the highest concentration causing a marked rise in late apoptotic cells. The other examined compounds had no apoptotic effect on this cell line (Figure 3I–L and Figure S5).

### 2.3. Cell Cycle Regulator Expression Profile

In this set of experiments, we measured the mRNA levels of 24 target genes as a screening to find potential biological connections between ER-related and off-target pathways as well. Thresholds for fold-change in gene expression can vary depending on the biological response and sensitivity of the cell line to treatment, which were primarily determined by the previously measured IC50 values. In this study, a 10-fold change threshold was used for PANC1 cells in response to T5 and T15, which are cytotoxic and have low IC50 values. These substances are highly potent, and small changes in gene expression can trigger significant biological effects, justifying the lower threshold. For T6, which induces G2/M arrest despite a higher IC50, a 50-fold change threshold was applied, as it requires larger changes in gene expression to significantly affect the cell cycle. For MCF7 and MDA-MB-231, which are less sensitive (higher IC50), a 50-fold change threshold was used. These cells show weaker responses to treatment, requiring larger changes in gene expression to be biologically meaningful.

**Figure 4:**
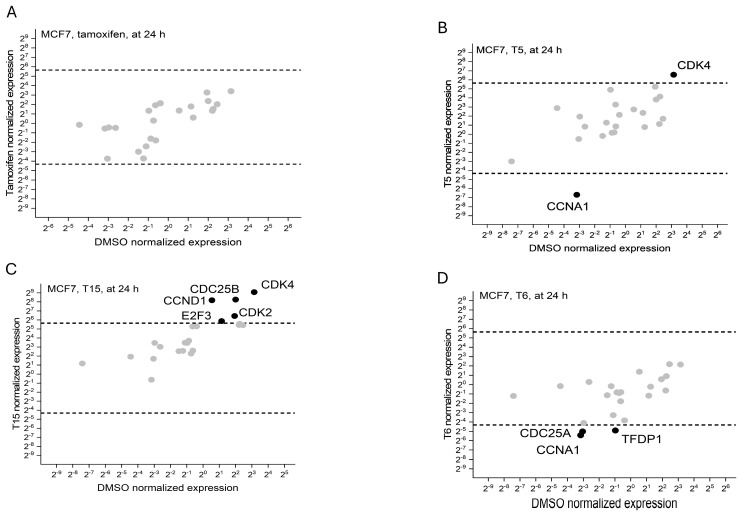
Impact of tamoxifen (**A**), T5 (**B**), T15 (**C**) and T6 (**D**) on the expression of cell cycle regulators on MCF7 cells. Cells were treated with 25 µM of the chosen derivative. DMSO was used as a control. Incubation before mRNA isolation was 24 h. Each result represents the measurement of two parallel samples (*n* = 2). Expression values of the derivative group and the DMSO were normalized to that of the housekeeping gene control. Up- or downregulation was recognized as significant when the number determined by the ratio of the normalized expression value in the group treated with the tamoxifen derivative and the normalized expression value in the DMSO-treated cells exceeded a 50-fold change, marked by the horizontal parallel reference lines. Normalization in both cases to the GAPDH housekeeping gene was carried out.

**Figure 5:**
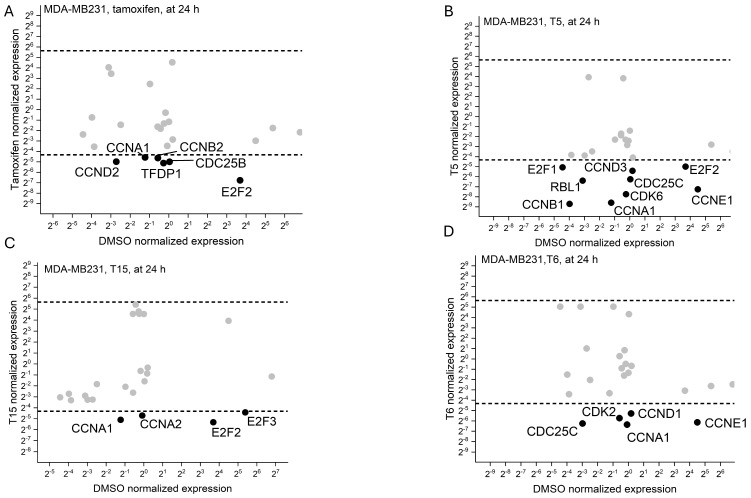
Impact of tamoxifen (**A**), T5 (**B**), T15 (**C**) and T6 (**D**) on the expression of cell cycle regulators on MDA-MB-231 cells. Cells were treated with 25 µM of the chosen derivative. DMSO was used as a control. Incubation was performed before mRNA isolation for 24 h. Each result represents the measurement of two parallel samples (*n* = 2). Expression values of the derivative group and the DMSO were normalized to that of the housekeeping gene control. Up- or downregulation was recognized as significant when the number determined by the ratio of the normalized derivative and the normalized DMSO value exceeded a 50-fold change, marked by the horizontal parallel reference lines. Normalization to the GAPDH housekeeping gene was carried out in both cases.

**Figure 6:**
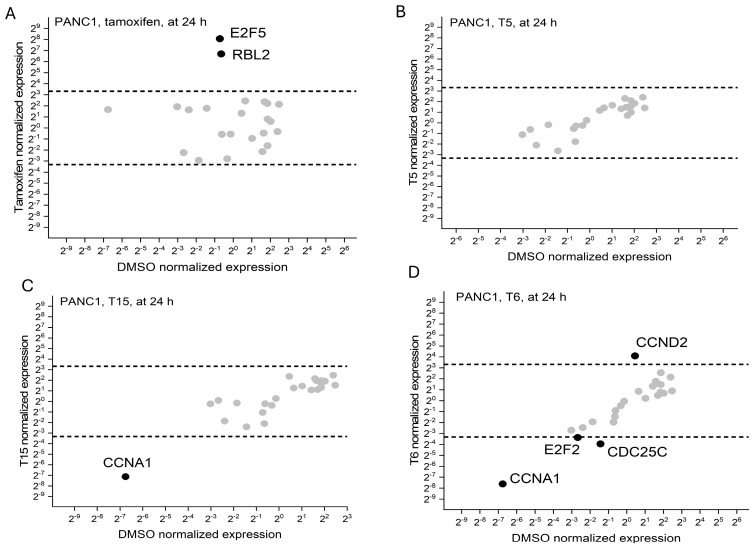
Impact of tamoxifen (**A**), T5 (**B**), T15 (**C**) and T6 (**D**) on the expression of cell cycle regulators on PANC1 cells. Cells were treated with 25 µM of the chosen derivative. DMSO was used as a control. Incubation was performed before mRNA isolation for 24 h. Each result represents the measurement of two parallel samples (*n* = 2). Expression values of the derivative group and the DMSO were normalized to that of the housekeeping gene control. Up- or downregulation was recognized as significant when the number determined by the ratio of the normalized derivative and the normalized DMSO value exceeded a 10-fold change, marked by the horizontal parallel reference lines. Normalization to the GAPDH housekeeping gene was performed in both cases.

Tamoxifen did not alter the expression of cell cycle regulators in MCF7 cells (Figure 4A). On MDA-MB231 cells, the expression of *CCNA1*, *CCNA2*, *CCNB2*, *CCND2*, *CDC25A*, *CDC25B* and *TFDP1* was lowered after tamoxifen treatment (Figure 5A). On PANC1 cells, only *E2F5* and *RBL2* were affected by tamoxifen, and higher levels were observed after 24 h of treatment (Figure 6A).

On MCF7 cells, T5 downregulated the expression of *CCNA1* but upregulated the expression of *CDK4* (Figure 4B). On MDA-MB231 cells, decreased levels of *E2F2*, *RBL1*, *CCNB1*, *CCND3*, *CDC25C*, *CDK6*, *CCNA1*, *E2F2* and *CCNA1* were observed upon exposure to T5 (Figure 5B). On PANC1 cells, no significant alterations were observed during the 24 h incubation period (Figure 6C).

T15 treatment on MCF7 cells led to the upregulation of *CCND1*, *CDC25B*, *E2F3*, *CDK2* and *CDK4* (Figure 4C). MDA-MB231 cells expressed less *CCNA1*, *CCNA2*, *E2F2* and *E2F3* following T15 treatment (Figure 5C). In PANC1 cells, T15 downregulated the expression of *CCNA1* (Figure 6C).

The indene-based T6 derivative decreased the levels of *CCNA1*, *CDC25A* and *TFDP1* after 24 h following treatment on MCF7 cells (Figure 4D). On MDA-MB231 cells, *CCNA1*, *CCND1*, *CCNE1*, *CDC25C* and *CDK2* were downregulated (Figure 5D). On PANC1 cells, *CCND2* was upregulated by T6; however, *CCNA1, CDC25C* and *E2F2* mRNA levels were decreased (Figure 6D).

### 2.4. Oxidative Stress Pathway Regulator Expression Profile

By summarizing our present and previous results on ROS-inducing activity of the tamoxifen derivatives, T15 proved to be the most potent promoter of ROS production in our experimental setup, so it was selected as the model compound for this part. Results regarding the ROS production elevating properties of tamoxifen and T15 were previously published by us [4], but the molecular background mechanism was not yet described in that publication. For this reason, expression levels of 91 regulators of the oxidative stress pathway were measured after tamoxifen and T15 treatment on all three cell lines. Depending on the treatment and the cell type, the expression profile of these regulators varied following the 24 h incubation period.

**Figure 7:**
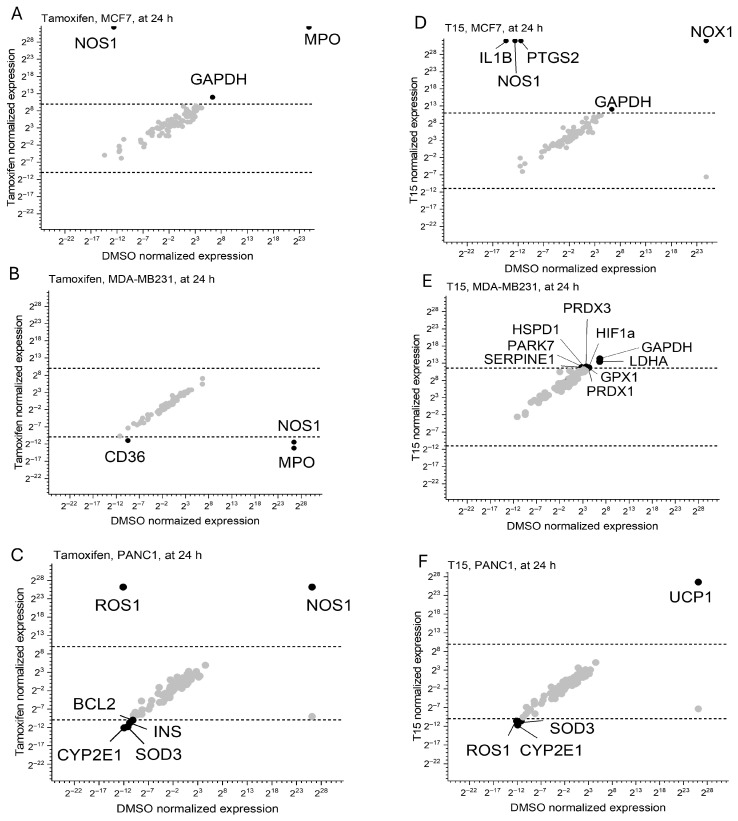
Impact of tamoxifen and T15 on the expression of oxidative stress regulators on MCF7 (**A**,**D**), MDA-MB-231 (**B**,**E**), and PANC1 (**C**,**F**) cells. Each cell line was treated with 25 µM of the chosen derivative. DMSO was used as a control. Incubation before mRNA isolation was 24 h. Each result represents the measurement of two parallel samples (*n* = 2). Expression values of the derivative group were normalized to those of the DMSO control. Up- or downregulation was recognized as significant when it exceeded a 1000-fold change, marked by the horizontal parallel reference lines. The significance threshold was set at this value to focus on the most biologically relevant genes, ensuring that only the most important changes are highlighted while avoiding consideration of smaller, potentially biologically irrelevant variations.

On MCF7 cells, tamoxifen treatment elevated the expression of *NOS1*, *GAPDH* and *MPO*. On MDA-MB231 cells, tamoxifen, however, downregulated the mRNA levels of *CD36*, *NOS1* and *MPO*. On PANC1 cells, *ROS1* and *NOS1* were upregulated; however, levels of *BCL2*, *INS*, *CYP2E1* and *SOD3* were decreased by tamoxifen compared to the DMSO control (Figure 7A–C).

Treatment with T15 upregulated the expression of *NOS1*, *NOX1*, *GAPDH*, *IL1B* and *PTGS2* on MCF7 cells. MDA-MB231 cells showed increased expression of *SERPINE1, PARK7*, *HSPD1*, *PRDX3*, *HIF1a*, *GAPDH*, *LDHA*, *GPX1* and *PRDX1* upon exposure to T15. On PANC1 cells, T15 elevated the mRNA levels of *UCP1* while downregulating the expression of *ROS1*, *SOD1* and *CYP2E1* (Figure 7D–F).

### 2.5. Apoptotic Regulator Protein Levels

Protein levels of 35 different regulators of the apoptotic process were measured on MCF7 cells after treatment with tamoxifen, T5, T15 and T6. This cell line was chosen as the experimental model for this measurement as it expresses all three major ER isoforms.

**Figure 8:**
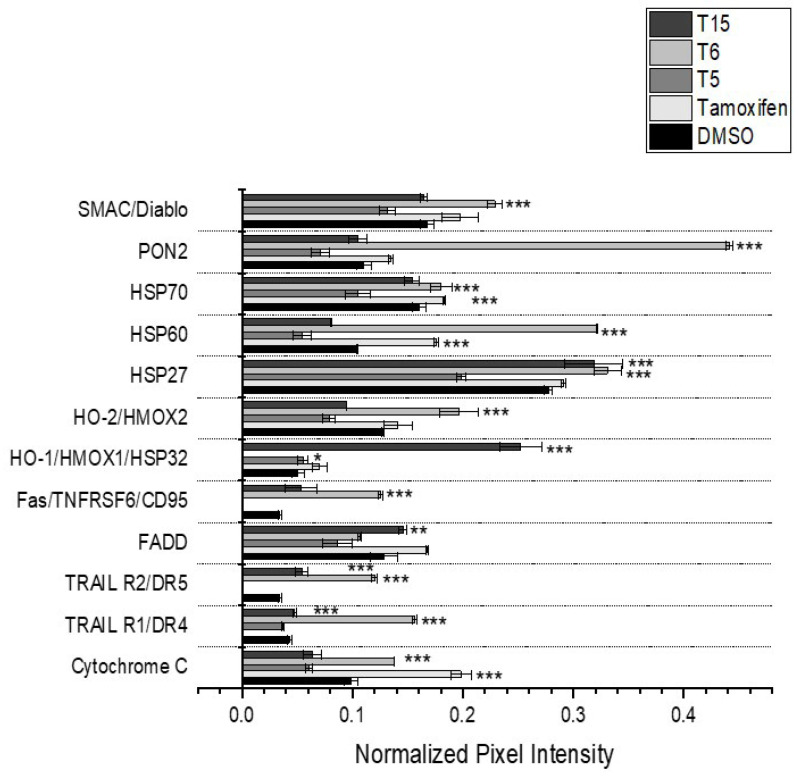
Impact of tamoxifen, T5, T6 and T15 on MCF7 cells regarding apoptotic regulator expression. Two parallel samples (*n* = 2) were measured after an incubation period of 24 h. Cells were treated with 25 µM. Data are given as mean values  ±  standard deviation (SD). Pixel intensity values were normalized to that of the internal reference points. Asterisks mark the subpopulation, where the normalized pixel intensity is significantly higher compared to the DMSO control (*p* < 0.05: *, *p* < 0.01: **, *p* < 0.001: ***) determined by the one-way ANOVA test followed by Fisher’s LSD post hoc test.

Tamoxifen treatment upregulated CytC, Hsp70 and Hsp 60, while downregulating the levels of DR4, DR5 and Fas. The ferrocene-linked T5 elevates the expression of HO1, but lower levels of CytC, DR4, DR5, FADD, Fas, PON2 and SMAC were observed following treatment. T15 treatment upregulates DR4, DR5, FADD, HO1 and Hsp27, while downregulating CytC, Hsp60, Hsp70, and SMAC. The indene-based T6 derivative elevates the expression levels of CytC, DR4, DR5, Fas, Hsp27, Hsp60, Hsp70, PON2 and SMAC but lowers FADD and HO1 protein levels (Figure 8, and for an easier look-through, it is also summarized in Table 2).

## 3. Discussion

In the present study, we summarize all the findings that help to understand the mechanisms underlying the increased antitumor effect of the ferrocene-linked drugs (T5 and T15) and the new, indene-based tamoxifen derivative (T6), on different cancer cell lines, modeling high-mortality tumor types.

Cell viability assays revealed that the inclusion of a ferrocene or an indene moiety enhances the antiproliferative impact across all examined cell lines. The underlying mechanism behind this favorable antitumor effect can be approached from two perspectives.

The chemical structures—most importantly the differences in the rotational freedom of the molecules—are a key factor in the antitumor effect of ferrocene-containing derivatives. Due to the rotational flexibility, they might bind differently to the various ER subtypes. We attribute these shifts partly to ligand conformational plasticity and rotational freedom introduced by the ferrocenyl moiety. Modern ER literature emphasizes that subtle changes in ligand geometry/dihedrals control helix-12 positioning and co-regulator recruitment, producing different transcriptional outputs and resistance phenotypes. This applies to SERM scaffolds such as 4-OHT and becomes even more relevant in the presence of ERα mutations that stabilize active-like helix-12 conformations [12,13].

The ferrocene unit itself contributes additional low-barrier cyclopentadienyl-ring rotational modes and acts as a conformational hinge when embedded in flexible linkers, broadening the set of ligand poses available in the ER pocket and, by extension, the signaling states that can be stabilized. Recent physical–organic and med-chem studies directly document ferrocene’s ring-rotation dynamics and its use as a dynamic linker element that modulates molecular folding and cell permeability—features consistent with the pose diversity we infer for T5 and T15 [14,15].

Previous studies have shown that not only tamoxifen but also its ferrocene-linked and halogenated [16,17,18] derivatives can bind to ER isoforms [18,19]. Thus, in ER-expressing cell lines, ER-related target genes may contribute to their anti-tumor actions. However, tamoxifen is also known to interact with several ER-independent pathways. To delve deeper into whether the compounds in this study favor the ER-dependent or ER-independent pathway, we characterized the estrogen receptor profile of our model cells, and subsequently, we assessed their impact on the cell cycle, oxidative stress, apoptosis and the expression of key regulators in these crucial pathways.

In our previous study, we confirmed that all three cell lines express ERα; however, only the MCF7 and MDA-MB-231 cell lines expressed ERβ. In PANC-1 cells, only GPER1 was detectable, while this receptor showed the highest expression in MCF7 cells.

Tamoxifen, categorized as a SERM, acts as either an agonist or antagonist on ER, depending on tissue type and the cellular ratio of the different isoforms [20]. While tamoxifen serves as an antagonist to estrogen receptors α and β in breast and pancreatic tissues, it acts as an agonist on GPER1 [21,22]. Throughout the synthesis of the T5 and T15 ferrocene-linked and the indene-based T6 derivatives, the foundational pharmacophore structure of the original tamoxifen was maintained. This preservation indicates that they retained the selective estrogen receptor modulator (SERM)-like property of tamoxifen and interacted with receptors in a manner akin to the parent molecule.

Cell cycle analysis was conducted to determine whether the compounds induce apoptosis or necrosis (resulting in the elevation of the subG1 population in the flow cytometry measurement) or induce cell cycle arrest. It should be noted that the concentration where each derivative was used was 25 µM. Derivatives only affected the progression of the cell cycle on cell lines where the IC50 value was higher than 25 µM; where it was lower, they increased the count in the subG1 group. We demonstrated that both T5 and T15 were capable of inducing cell cycle arrest in the G1 phase of breast cancer cells [4]. Treatment with the indene-based T6 compound led to a G2/M phase arrest on the PANC1 pancreatic cancer cell line, while the treatment with this compound resulted in a significant elevation in the subG1 population of the MCF7 breast cancer cells, containing the apoptotic bodies and necrotic debris of cells.

The incorporation of an indene moiety into tamoxifen analogues enhances their anticancer properties through several key mechanisms. Firstly, replacing the C-phenyl ring of tamoxifen with an indole or oxindole scaffold improves the compound’s binding affinity to ERα, thereby enhancing its antagonistic effect on estrogen-driven proliferation. Secondly, it might increase the activation of caspase-8 and the induction of apoptosis [23]. On the MCF7 cell line, which proved to be the most sensitive to T6, this result was indeed observed, with apoptosis-inducing effects detectable as early as 24 h.

The phases of the cell cycle are regulated by the different ER subtypes, whose roles depend on the exact phase. ERα and ERβ act as general mitogen stimuli and play a crucial role at the very beginning of the G1 phase, leading to the assembly of the CDK4/6-Cyclin D complex [24]. They also serve as an expressional starting point in the positive feedback loop found at the transition from the G1 to the S phase, through the CDK2-Cyclin E complex [19]. Expression of some other key players in the cell cycle, such as cyclin A, cyclin E and EF2, are dependent on the nuclear ER subtypes [25,26,27]. GPER1 regulates the S and G2/M phases, mainly through the activation of the MAP/Erk pathway [28] and the promotion of the microtubule assembly [29,30].

Tamoxifen’s primary action is through the nuclear subtypes of ER. In MCF7 cells, tamoxifen had minimal impact on cell cycle regulators; however, it induced an S phase arrest. On PANC1 cells, tamoxifen increased the expression of E2F5 and RBL2, but this shift in regulators did not result in a cell cycle arrest. In MDA-MB-231 cells, tamoxifen significantly downregulated key regulators such as CCND2 and TFDP1, both of which result in a G1 phase arrest [31,32]. This suggests that tamoxifen can also induce G1 arrest via the nuclear ER subtypes, the expressional regulators of these two proteins [33,34].

The T5 and T15 derivatives, both based on ferrocene, exhibited strong effects on cell cycle regulation, particularly in the G1 phase. T5 treatment led to the downregulation of CCNA1 and the upregulation of CDK4, suggesting that this is why it may inhibit G1 phase progression [35]. In MDA-MB-231 cells, T5 induced downregulation of several cyclins and CDKs, pointing to a potential G1 arrest. In PANC1 cells, no significant changes were observed regarding the cell cycle, suggesting that such effects of T5 are more pronounced in cell lines expressing the nuclear ER subtypes [36].

T15, the other ferrocene-based compound, upregulated CCND1, CDC25B, E2F3, CDK2 and CDK4 in MCF7 cells, which are regulators of the G1/S transition [37]. However, this expression pattern does not explain the observed G1 arrest in MCF7 cells. There might be other factors in play that were not measured by our set of experiments. CDK inhibitors such as p21, p27 and p16 might inhibit the activity of the Cyclin D-CDK4/6 and CDK2 complexes, even if they are upregulated, thus preventing Rb phosphorylation and blocking progression into the S phase [38,39]. Furthermore, alterations in the Rb/E2F pathway could prevent the activation of E2F transcription factors despite the presence of Cyclin D and CDK complexes, thereby causing a G1 arrest [40]. Additionally, activation of DNA damage or stress-induced checkpoints may further inhibit CDK activity, contributing to G1 phase arrest [41]. In MDA-MB-231 cells, T15 decreased the expression of key cyclins and E2F transcription factors, indicating G1 arrest. These effects are consistent with the known ability of ferrocene derivatives to regulate the G1/S checkpoint [42,43].

The T6 derivative, based on an indene structure, induced a G2/M arrest in PANC1 cells, while it had no effect on the cell cycle progression on the other two cell lines, mainly expressing the nuclear ER subtypes. In PANC1 cells, T6 upregulated CCND2 but decreased the expression of CCNA1, CDC25C, and E2F2, suggesting a dual-phase effect on the cell cycle, promoting G1 progression while inducing G2/M arrest. This dual-phase regulation by T6 is consistent with literature showing that indene-based compounds can regulate both G1 and G2/M checkpoints [44]. The ability of T6 to modulate both phases of the cell cycle highlights its potential as a dual-phase cell cycle regulator [45]. As PANC1 is a mainly GEPR1-expressing cell line, we suspect that the T6 indene-based tamoxifen derivative favors this ER isoform regarding cell cycle regulation, leading to its activation and the decreased transcription of CDC25C and a G2/M phase arrest [46].

ROS production elicited by tamoxifen and its ferrocene-linked derivatives were already measured, and we were keen to explore how the indene-based derivative with specific conformational flexibility compares to these molecules. Contrary to expectations from the literature, T6 showed a strong oxidative potential on MCF7 cells; however, only at the highest concentration. Based on our overall results, we chose the ferrocene-linked T15 for exploring the oxidative stress regulator expression profile, as this one showed the most potent and widespread oxidative stress-inducing capability on our cell lines. We hypothesized that there is a basic difference between the molecular background of the ROS production-promoting capability of tamoxifen and its ferrocene-linked derivative, T15, depending on the cellular model.

On the mainly Erα-expressing MCF7 cells, tamoxifen acts through the higher expression of myeloperoxidase (MPO) and nitric oxide synthase 1 (NOS1), which are both Erα-induced targets [47,48]. On the highly ERβ-positive MDA-MB231 cells, tamoxifen treatment resulted in the downregulation of the same NOS1 and MPO isoforms, while CD36 was also downregulated. The downregulation of MPO seemingly has an anti-inflammatory effect that inhibits the growth of cancer cells, which aligns with the results of our cell viability measurements but does not explain the higher ROS production observed upon treatment. Stimulation of ERβ promotes this effect on an expressional level [49]. NOS1 expression is also regulated by this ER isoform [50]. The signaling mediated by ligands of the scavenger receptor CD36 typically triggers the production of intracellular ROS. CD36 attenuates angiogenesis by binding to thrombospondin-1 (TSP-1) and thereby inducing apoptosis or blocking the vascular endothelial growth factor receptor 2 pathway in tumor microvascular endothelial cells. There is no clear evidence that CD36 would be an ERβ target gene. Other indirect mechanisms, such as the peroxisome-proliferator-activated receptor (PPAR) activation, might explain the observed effects. The inhibition of ERβ by tamoxifen leads to the deactivation of PPARγ through transcriptional crosstalk, direct interaction and also ligand influence [51]. Tamoxifen also increases the phosphorylation of PPARγ via the activation of Erk1/2. Sufficient levels and regulatory activity of the PPARγ play a critical role in the expression of CD36 [52]. On PANC1 cells, tamoxifen treatment upregulated the expression of ROS1 and NOS1. There is no direct evidence that these mediators would be directly regulated by the predominant GPER1 isoform of this cell line. Other, indirect connections, such as the off-target inhibition of protein kinase C (PKC), might play a role in the observed effect, as PKC, GPER1 and ROS1 have the same downstream signaling pathways. The inhibition of one might lead to the compensatory overexpression of the other [53,54]. Tamoxifen, on the other hand, downregulated BCL2, CYP2E1 and superoxide dismutase 3 (SOD3). In addition to its well-known anti-apoptotic function, Bcl-2 is involved in regulating mitochondrial ROS by influencing mitochondrial complex IV activity, promoting the incorporation of glutathione (GSH) into mitochondria, and interacting with the small GTPase Rac1 at the mitochondrial level [55]. CYP2E1, one of the most active isoforms of the CYP450 family, has been shown to produce intracellular ROS, which can be linked to cancer development and metastasis [56]. CYP2E1 inhibition decreases intracellular ROS accumulation and inhibits cell cycle progression in in vitro conditions by controlling the levels of SOD3 [20,57].

T15 can likely interact with the different estrogen receptor isoforms, as it retains the same pharmacophore region as the parent molecule. Elevated levels of *NOS1* after T15 treatment on MCF7 cells and the repressed expression of *SOD3* and *CYP2E1* on PANC1 cells could be the result of this receptor interaction. Excessive ROS production by T15 treatment activates different counter mechanisms trying to partially protect the tumor cells against oxidative stress, such as the higher expression of NOX1 on MCF7 cells; PARK7, PRDX1 and PRDX3 on MDA-MB231 cells; and UCP1 elevation in PANC1 cells.

Ferroptosis is a distinctive form of cell death driven by iron-dependent phospholipid peroxidation. The incorporation of a ferrocene moiety into different therapeutic agents enhances the possibility of this special kind of cell death [58,59,60,61,62]. The promising antitumor activity of T5 and T15 makes it worth investigating whether they induce ferroptosis as a future research aim.

One of the ROS regulators that showed high elevation upon T15 treatment was NOX1. It has been shown that NOX1 generates ROS in neurons by catalyzing the reaction between NADPH and oxygen molecules. Additionally, overexpression of NOX1 promotes autophagy associated with ferroptosis (ferritinophagy) [61]. It is assumed that, by activating the NOX1 pathway, T15 could contribute to promoting oxidative stress and lipid peroxidation [63].

Parkinson’s protein 7 (PARK7), which was significantly induced by T15 in MDA-MB231, is widely expressed and plays a variety of roles in pathological conditions. PARK7 directly combats oxidative stress by undergoing oxidation at its Cys106 residue. Additionally, it prevents ferroptosis by modulating the transcription of genes involved in lipid metabolism, ROS regulation, and iron homeostasis. PARK7 also stabilizes Nrf2, a key transcription factor that regulates the antioxidant response, further enhancing the cell’s defense against oxidative damage and ferroptosis. Conversely, when ROS levels rise excessively, Nrf2 can initiate various forms of programmed cell death, including ferroptosis. [64].

In close connection with the above-mentioned ROS production and a possible ferroptosis-inducing capability, the novel tamoxifen derivatives also activate the classical routes of apoptosis. The ferrocene-linked agents (T5 and T15) have a stronger cell-viability-decreasing effect on PANC1 and MCF7 cells compared to tamoxifen, while the indene-based T6 derivative had the best IC_50_ value on MCF7 cells. All three compounds also induce apoptosis in our model cells. The increase in both the early and late phases of apoptosis was observed, depending on the cell type and the concentration of the agents, but through a possibly different molecular mechanism of action. To test the hypothesis, we chose the MCF7 cell line, as it expresses all three major estrogen receptor isoforms, and performed experiments on it to measure the expression of apoptotic regulator proteins.

When apoptotic mechanisms are activated, cytochrome c is released from mitochondria into the cytosol. In this new location, it either initiates the caspase cascade within the intrinsic apoptotic pathway or contributes to the amplification of extrinsic apoptotic signaling [65]. GPER1 activation leads to intracellular release of cytochrome C [66], which was observed upon treatment of the MCF7 cells with tamoxifen and the indene-based T6 derivative. However, the ferrocene-linked derivatives decreased the level of cytochrome C compared to the non-treated sample.

Heat shock proteins Hsp27, Hsp60 and Hsp70 are molecular chaperones whose expression levels rise in response to various stressors. These proteins play a protective role, assisting cells in surviving potentially fatal conditions [67]. The inducible isoform, heme oxygenase-1 (HO1), also plays a pivotal role in cellular defense [68]. Since these two kinds of proteins connect the oxidative stress to programmed cell death, we hypothesize that the observed increase in their expression level is a compensatory response elicited by the ROS production of the tamoxifen derivatives.

The drugs investigated in this article not only affect the previously described common regulators of the intrinsic apoptotic pathway, but they also influence the extrinsic pathway. We can hypothesize a mechanism of action based on the effects of tamoxifen derivatives on the expression of the extrinsic apoptotic pathway. Death receptors, members of the tumor necrosis factor (TNF) receptor superfamily, are located on the cell surface and become activated upon binding to specific ligands. Their activation triggers a signaling cascade culminating in the formation of the Death-Inducing Signaling Complex (DISC) with the help of the intracellular death domain (DD) of the receptor and adaptor proteins like the Fas-associated protein with death domain (FADD). DISC recruits and activates initiator caspases [69,70]. Tamoxifen decreases the expression of death receptor 5 (DR5) through different possible mechanisms. DR5 expression can be influenced by the estrogen receptor signaling pathway. By acting as an antagonist, tamoxifen reduces the transcription of DR5 [52]. DR5 expression is also regulated by transcription factors such as NF-κB and p53, and as an off-target effect, tamoxifen can modulate these pathways, too. Interference with them potentially leads to DR5 downregulation [71,72]. On the other hand, the ferrocene-linked T15 and the indene-based T6 derivative elevated the levels of the players of the extrinsic apoptotic pathway. Ferrocene-linked tamoxifen derivatives introduce a redox-active moiety. Elevated oxidative stress can activate transcription factors like C/EBP homologous protein (CHOP), which are known to upregulate DR5 expression. The incorporated ferrocene moiety makes the derivative a more potent inducer of endoplasmic reticulum stress compared to tamoxifen. This leads to the activation of the unfolded protein response (UPR). A key component of the UPR is the transcription factor CHOP [73]. Both the introduction of ferrocene and modifications in the conformational flexibility may also sensitize cancer cells to TNF-related apoptosis-inducing ligand (TRAIL), which is mediated through DR5 [74].

While the aim of this article was to characterize the derivatives from an in vitro experimental point of view, we would have liked to look into, whether there are studies with ferrocene-modified or indene-based tamoxifen analogues moving towards in vivo experiments. As of today, no ferrocene- or indene-based tamoxifen derivatives are in clinical trials or FDA-approved. Tamoxifen family agents that have reached the clinic include endoxifen, investigated in Phase II/III trials (ongoing, ClinicalTrials.gov ID: NCT02311933), and older analogs like idoxifene [75]; however, these were ultimately discontinued. Preclinical studies with ferrocene or indene-containing tamoxifen analogues are listed in Table 3 and Table 4, respectively. Contrary to ferrocene-modified analogues, which remained a recurring topic, indene-based ER ligands largely fell off the radar after early structure–activity studies in the 1980s–2000s. Since then, research efforts and FDA clinical trials have shifted away from indene scaffolds toward more potent and clinically viable approaches, including oral selective estrogen receptor degraders (SERDs) [76], selective estrogen receptor covalent antagonists (SERCAs) [77] and PROTAC degraders [78]. We believe the novelty of our work lies in the repositioning of the scaffold for next-generation ER antagonism and resistance-aware therapy on a broad spectrum of common malignancies.

## 4. Materials and Methods

### 4.1. Synthesis of the Novel Tamoxifen Derivatives

The synthesis and detailed chemical characterization of the indene-based T6 tamoxifene analogue have been reported in a previous publication [16], while the ferrocene-linked derivatives were also previously described by our research group [4].

### 4.2. Cell Lines and Culturing

The effects of tamoxifen and its derivatives were investigated on three cell lines, which were obtained from the European Collection of Authenticated Cell Cultures (ECACC, Salisbury, UK). All cell cultures were maintained at 37 °C in an incubator with a humidified atmosphere of 5% CO_2_. In every case, the base medium was supplemented with 10% FCS (Lonza Group Ltd., Basel, Switzerland), L-glutamine (2 mM) and 100 μg/mL penicillin/streptomycin (Gibco^®^/Invitrogen Corporation, New York, NY, USA). Mycoplasma testing was performed with PCR regularly on all cell lines from the supernatant.

PANC1 (87092802 ECCAC) is a pancreatic adenocarcinoma cell line established from a pancreatic carcinoma of ductal origin from a 56-year-old Caucasian male. This cell culture was maintained in the supplemented Dulbecco’s Modified Eagle Medium (Lonza Group Ltd., Basel, Switzerland).

MCF7 (86012803 ECCAC) is an ER-positive breast adenocarcinoma cell line established from the pleural effusion of a 69-year-old Caucasian female. MCF7 cells were cultured in supplemented Minimum Essential Medium Eagle (Sigma-Aldrich, St. Louis, MO, USA), also containing 1% Non-Essential Amino Acid (Sigma-Aldrich, St. Louis, MO, USA).

MDA-MB-231 (92020424 ECCAC) is an ER-negative breast adenocarcinoma cell line also established from pleural effusion. Culturing of this cell line was performed with supplemented Dulbecco’s Modified Eagle Medium (Lonza Group Ltd., Basel, Switzerland).

NHDF cells (C-12300 Sigma-Aldrich) are isolated from the dermis of juvenile foreskin or adult skin from different locations. Culturing of this cell line was performed with supplemented Dulbecco’s Modified Eagle Medium (Lonza Group Ltd., Basel, Switzerland).

### 4.3. Cell Viability Assays

The xCELLigence SP system (Agilent, Santa Clara, CA, USA) is an impedance-based method that allows real-time monitoring of different cell biological properties, such as a quantitative readout of cell number, proliferation rate, cell size and shape, and cell-substrate attachment quality. Adherent cells, such as PANC1 and MCF7, attach to the bottom of the wells and disrupt the electric current between the microelectrodes found on the bottom, which generates an increased impedance signal higher than the background signal of the system. Upon treating the cells with T6, the cells acquire a round shape and detach from the bottom, which restores the electric current between the microelectrodes, and with it, the measured impedance value decreases. A relative and dimensionless parameter, the cell index (CI), can be calculated from the detected impedance change by the following formula: CI = (Zi−Z0)/F where Zi is the impedance at a given time point, Z0 is the impedance at t = 0 h, and Fi is a constant depending on the frequency (F10kHz = 15). To allow real-time measurement, the system was placed in an incubator at 37 °C with a humidified atmosphere of 5% CO_2_. First, a background measurement was performed to acquire a baseline impedance curve by adding 100 μL of complete culture medium to each well and recording the CI for 1 h. Next, the cells were added to the wells at a concentration of 10,000 cells/well and cultured for 24 h. Upon completion of 24 h incubation, cells were treated with tamoxifen or its derivates. CI values were measured for a further 72 h. Identical points of the concentration course study refer to the average of 3 parallel measurements. For comparison of the antitumor activity, the IC_50_ values were calculated from the Delta CI values of each concentration obtained at 72 h with the RTCA 2.0 software (ACEA Biosciences, San Diego, CA, USA).

The viability of MDA-MB-231 cells after treatment with T6 was determined by AlamarBlue assay (Thermo Fisher Scientific, Watham, MA, USA) according to the manufacturer’s instruction at 72 h following treatment. Cells were seeded at a 10,000/well concentration. After 4 h of incubation with the fluorescent reagent, the fluorescence intensity was measured with a LS-50B Luminescence Spectrometer (Perkin Elmer Ltd., Buckinghamshire, UK). Each data point represents the average of three parallel measurements. IC_50_ values were calculated at 24, 48 and 72 h following treatment.

To determine the IC_50_ value of derivatives, a dilution series of 9 concentrations (250 nM–100 µM) was made. Cell culture medium and DMSO (<1 vol%) were used as controls. The IC_50_ value of the tested compounds was determined by fitting a sigmoidal dose–response curve to the data.

### 4.4. Cell Cycle Analysis

FACSCalibur flow cytometer (Becton Dickinson, San Jose, CA, USA) was used to measure the DNA content of cells with propidium iodide (Sigma-Aldrich, St. Louis, MO, USA) intercalated stoichiometrically to the double-stranded DNA.

The cells were seeded in 12-well plates at a concentration of 250,000 cells/mL. After 24 h of incubation, they were treated with T6 at a concentration of 25 µM, as this was the EC_80_ value for tamoxifen at 72 h (i.e., the concentration where 80% of the cells are viable following treatment) on PANC1 cells. Cells were then harvested after 24 and 48 h of treatment and were fixed in ice-cold 70% ethanol and kept at −20 °C for 24 h.

The samples were centrifuged and resuspended in RNase (100 μg/mL, Sigma-Aldrich, St. Louis, MO, USA) containing citric acid/sodium phosphate buffer (pH = 7.8). Propidium iodide was added to the sample at 10 μg/mL concentration right before the flow cytometric measurements, collecting 25,000 cells/sample. Data were analyzed by CellQuest Pro (Becton Dickinson, San Jose, CA, USA) and Flowing 2.5.1 (Turku Centre of Biotechnology, Turku, Finland) software. For aggregate and debris discrimination, the FL2-Width vs. FL2-Area plot was used, and the gated cells are displayed in the FL2-Area histogram to assign percentage values to each population of cell cycle stages. Statistical analysis was performed with MS Excel and OriginPro 2018 (OriginLab Corporation, Northampton, MA, USA).

### 4.5. ROSGlo Assay

Cells were seeded in a white-walled, clear-bottom 96-well plate at a concentration of 10,000 cells/well, in 70 μL culture medium. After 24 h of incubation at previously described conditions, each well was treated with 10 μL of T6 at a concentration of 0.5, 2.5 and 25 μM. Culture medium and DMSO (<1 vol%) were used as controls, and 600 nM of Bortezomib was used as a positive control. Each data point represents the average of 3 parallel measurements. Components of the ROSGlo assay (Promega, Southhampton, UK) were prepared and added according to the manufacturer’s instructions. Luminescence was measured 24 h upon treatment with Fluoroskan^TM^ FL Microplate Fluorometer and Luminometer (Thermo Scientific, Waltham, MA, USA).

### 4.6. Measuring Apoptosis Induction

Cells were seeded in 24-well plates at a concentration of 100,000 cells/well. After 24 h of incubation, they were treated with tamoxifen, T5, T15 and T6 at a concentration of 0,5, 2,5 and 25 µM of the chosen compound. Cells were then harvested after 24 h of treatment and were resuspended in Annexin Binding Buffer (Thermo Scientific, Waltham, MA, USA, Catalog number: V13246) and labeled with either FITC-conjugated Annexin V (Thermo Scientific, Waltham, MA, USA, Catalog number: A13199) or 7-aminoactinomycin D (7AAD—Thermo Scientific, Waltham, MA, USA, Catalog number: A1310). Labeled samples were then incubated at room temperature for 10 min in a container isolated from light. FACSCalibur flow cytometer (Becton Dickinson, San Jose, CA, USA) was used to measure the signal intensity from both dyes. Data were analyzed by CellQuest Pro (Becton Dickinson, San Jose, CA, USA) and Flowing 2.5.1 (Turku Centre of Biotechnology, Turku, Finland) software. Statistical analysis was performed with MS Excel and OriginPro 2018 (OriginLab Corporation, Northampton, MA, USA).

### 4.7. Characterizing the Expression of Apoptosis Regulating Proteins

MCF7 cells were seeded in Petri dishes in 10 mL of culture medium at a concentration of 1.5 million cells/dish. After 24 h of incubation in the previously described conditions, cells were treated with 25 uM of tamoxifen, T5, T15 or T6. Culture medium and DMSO (<1 vol%) were used as controls. Twenty-four hours after treatment, cells were transferred to Eppendorf tubes and were washed with PBS and centrifuged at 1000× *g* at room temperature for 5 min. Lysis Buffer 17 (R&D Systems, Minneapolis, MN, USA) was added to each tube, and after 20 min of incubation, the tubes were centrifuged at 20,000× *g* at 4 °C for 20 min. The supernatant, containing the isolated proteins, was kept as aliquots at −70 °C until use. Protein concentration was measured using the Pierce™ BCA Protein Assay Kit (Thermo Fisher Scientific, Watham, MA, USA). The Proteome Profiler Human Human Apoptosis Array Kit membranes (R&D Systems, Minneapolis, MN, USA) were prepared with 200 μg of protein according to the manufacturer’s instructions. Membranes were exposed for 20 min to X-rays before reading chemiluminescence in the ChemiDoc XRS+ system (BioRad, Hercules, CA, USA). Image data were analyzed with the ImageLab 6.0.1 software (BioRad, Hercules, CA, USA). Each data point represents an average of two parallel measurements. The obtained mean pixel density was normalized with the mean of the reference spots for each membrane separately. A protein was only recognized as an expressed one if its normalized pixel intensity reached 20% of the mean pixel intensity of the reference spots.

### 4.8. Measuring the Expression of Cell Cycle and Oxidative Stress Regulating Factors

The change in the expression of regulating factors upon treatment was determined by real-time quantitative PCR after mRNA isolation and cDNA preparation.

Cells were seeded to 12-well, uncoated, clear-bottom plates at a concentration of 200,000 cells/well in 1 mL of culture medium. After 24 h of incubation, cells were treated with T6, T5, T15 or tamoxifen at a final concentration of 25 μM. Culture medium and DMSO (<1 vol%) were used as negative controls. After 24 h, mRNA was isolated from the treated cells by the Quiagen RNeasy Mini Kit (Quiagen Sciences, Germantown, MA, USA) according to the manufacturer’s manual. The total mRNA concentrations were measured with the NanoDrop 1000 UV-Visible spectrophotometer (Thermo Scientific, Waltham, MA, USA). Then cDNA was prepared with the SensiFAST cDNA Synthesis Kit (Meridian Bioscience, Cincinnati, OH, USA) according to the manufacturer’s protocol.

For mRNA expression, the predesigned Biorad Oxidative Stress Tier 1 H96 and Biorad Cell Cycle Generic H96 plates (Biorad Laboratories, Hercules, CA, USA) were used. Plates were prepared according to the manufacturer’s manual, with each well containing 10 nmol cDNA. Experiments were performed in triplicate, with each one originating from a different biological sample, seeded at the same time. Plates were then measured with the Biorad CFX 96 Touch Real-Time PCR Detection System (Biorad Laboratories, Hercules, CA, USA).

For later statistical analysis, three housekeeping genes—*GAPDH*, *TBP* and *HPRT*—were tested for their expression with the method described above. For further use, *GAPDH* was selected for the apoptosis experiments, as it provided the smallest Cq value or, in other words, the highest basal expression in the environment where the sample was treated with the DMSO only. *TBP*, the gene with the second smallest Cq value was used for the ROS production assay, as the array we used already contained *GAPDH* as a target gene.

### 4.9. Statistical Analysis

For statistical analysis, Origin Pro 8.0 (OriginLab Corporation, Northampton, MA, USA) and MS Excel software were used. One-way analysis of variance (ANOVA) followed by Fisher’s Least Significant Difference (Fisher’s LSD) post hoc test was performed. A *p*-value less than 0.05 was considered statistically significant (*p* < 0.05: *, *p* < 0.01: **, *p* < 0.001: ***). The experiments were performed in triplicate (*n* = 3) if not otherwise stated, and the data are presented as mean ± standard deviation (SD).

## 5. Conclusions

Previous series of experiments performed by our research group have already proved that the added ferrocene moiety decreases the cell viability compared to treatment with tamoxifen on breast and pancreatic cancer cell lines. The newly introduced T6 indene-based molecule with modified conformational flexibility also has a lower IC_50_ value than its parent molecule and has an excellent overall antitumor effect on the MCF7 cell line, which resembles the classical tumor type that tamoxifen is originally indicated for in a clinical setting. These effects are due to the drugs’ capability of inducing apoptosis, increasing ROS production and arresting the cell cycle. Additional research revealed several possible molecular mechanisms explaining the different effects elicited on the different ER isoform-expressing model cells. On nuclear ER isoform-expressing cells, receptor activation will lead to apoptosis induction through higher expression of the extrinsic apoptotic pathway. In the presence of GPER, apoptosis goes through the increased presence of cytochrome C. All the derivatives investigated, but especially the ferrocene-linked ones, were capable of increasing ROS production. The molecular mechanism behind this is diverse and independent of the ER expression, but a unique form, ferroptosis, might play a role here. These in vitro studies suggest that modifying tamoxifen with ferrocene and a relatively rigid indene moiety carrying hydroxyphenyl group produce substances with a potential to be far more effective against a wider range of tumor types than their parent molecule. Moreover, the structural versatility of the indene scaffold allows for fine-tuning of pharmacokinetic and pharmacodynamic properties, paving the way for the development of more effective and selective breast cancer therapeutics.

## Figures and Tables

**Table 1:** Comparison of the IC_50_ values of tamoxifen, T5, T15 and T6 on MCF7, MDA-MB-231 and PANC1 cells at 72 h after treatment. IC_50_ results are given in micromoles per liter (µM). Tamoxifen, T5 and T15 measurements were carried out previously and are already published by us [4]. They are again referenced here to serve a comparative purpose. Each cell line was treated with concentrations between 250 nM and 100 µM. All experiments were performed in triplicate (*n* = 3).

	MCF7	MDA-MB231	PANC1	NHDF
	IC_50_ at 72 h (µM)			
Tamoxifen	42.7	21.8	33.8	110.0
T5	43.3	26.3	12.5	243.5
T15	23.0	23.8	15.1	n.d.
T6	4.9	64.1	51.8	263.4

n.d. not detectable.

**Table 2:** Summary of the impact of tamoxifen, T5, T6 and T15 on MCF7 cells regarding apoptotic regulator expression. Expression change is given related to the DMSO control group.

Target Name	Tamoxifen	T5	T15	T6
SMAC/Diablo	no change	downregulation	downregulation	upregulation
PON2	no change	downregulation	no change	upregulation
HSP70	upregulation	no change	downregulation	upregulation
HSP60	upregulation	no change	downregulation	upregulation
HSP27	no change	no change	upregulation	upregulation
HO-2/HMOX2	no change	no change	no change	upregulation
HO-1/HMOX1/HSP32	no change	upregulation	upregulation	downregulation
Fas/TNFRSF6/CD95	downregulation	downregulation	no change	upregulation
FADD	no change	downregulation	upregulation	downregulation
TRAIL R2/DR5	downregulation	downregulation	upregulation	upregulation
TRAIL R1/DR4	downregulation	downregulation	upregulation	upregulation
Cytochrome C	upregulation	downregulation	downregulation	upregulation

**Table 3:** Summary of preclinical studies regarding ferrocene-based tamoxifen derivatives.

Molecule	Reference	Tumor Type/Model	Result
Fc-OH-TAM (ferrocenyl-4-hydroxytamoxifen; “ferrocifen”)	[79]	Breast cancer cell lines (MCF-7, T-47D, ZR-75-1; MDA-MB-231, SKBR-3, Hs578T)	Antiproliferative in ER+ and ER− cells; S-phase recruitment; ROS; ER-independent effects.
Hydroxyferrocifens	[80]	Breast cancer cell lines (MCF-7, MDA-MB-231)	Active on hormone-dependent & -independent lines; SERM-like antiproliferative effects.
Hydroxyferrocifens (cyclodextrin complexes)	[81]	Breast cancer cell lines	Improved formulation/solubility; strong in-vitro antiproliferative activity.
Fc-OH-TAM LNCs (“stealth” lipid nanocapsules)	[82]	TNBC xenograft (MDA-MB-231-luc, mouse)	Two 20 mg/kg i.p. doses delayed tumor growth; S-phase arrest preserved in LNCs.
Fc-diOH (ferrociphenol)	[83]	Intracranial 9L rat gliosarcoma	longer survival
Fc-diOH-LNCs with NFL peptide (targeting)	[84]	Glioma/GBM cells (U87MG)	NFL-peptide coating increased glioma-cell uptake vs. conventional LNCs.
P722 ferrocifen in LNCs	[85]	B16F10 melanoma (mouse)	Slower tumor growth, improved survival; intrinsic apoptosis and CD8+ T-cell activation.
P53 & P722 ferrocifens (stealth LNCs) ± chemo	[86]	Ovarian adenocarcinoma PDX (High/Low OXPHOS)	P722-LNCs + carboplatin/paclitaxel reduced tumor burden; P722 highlighted as lead.
Ferrociphenol/ferrocifen “next-gen” designs	[87]	Multiple cancer cell lines (in vitro)	New ferrociphenols with diverse reactive metabolites; sub-µM antiproliferative activity.
Hydroxyferrocifens → TrxR targeting (mechanism)	[88]	Mechanistic/enzymatic + cancer cell assays	Ferrocenyl quinone-methides potently inhibit TrxR; supports redox-based cytotoxicity.

**Table 4:** Summary of preclinical studies regarding indene-based tamoxifen derivatives.

Molecule	Reference	Tumor Type/Model	Result
2,3-Diarylindenes (hydroxylated)	[89]	ER binding (tool compounds)	ER binding measured; orientation considerations for indene ER ligands.
1,3- & 2,3-Diarylindenes (ERα/ERβ)	[90]	MCF-7	High-affinity ER binding; subtype-selective agonism/antagonism; inhibited E2-stimulated growth.
2,3-Disubstituted indenes	[91]	MCF-7	Weak steroid-receptor binding; modest/weak antiproliferative effects.

## Data Availability

The original contributions presented in this study are included in the article/Supplementary Materials. Further inquiries can be directed to the corresponding author(s).

## Supplementary Materials

The following supporting information can be downloaded at https://www.mdpi.com/article/10.3390/ph18091417/s1. Figure S1: Impact of T6 on the cell cycle at 24 h following treatment. Figure S2: Flow cytometry output for the cell cycle analysis. Experiments were performed in duplicate. For the sake of better representation, results from only one of the parallel samples are represented. Figure S3: Flow cytometry output for the apoptosis measurements on MCF7 cells. Experiments were performed in duplicate 24 h after treatment. For the sake of better representation, results from only one of the parallel samples are represented and where both the Annexin V and the 7AAD stains were used. Figure S4: Flow cytometry output for the apoptosis measurements on MDA-MB231 cells. Experiments were performed in duplicate 24 h after treatment. For the sake of better representation, results from only one of the parallel samples are represented and where both the Annexin V and the 7AAD stains were used. Figure S5: Flow cytometry output for the apoptosis measurements on PANC1 cells. Experiments were performed in duplicate 24 h after treatment. For the sake of better representation, results from only one of the parallel samples are represented and where both the Annexin V and the 7AAD stains were used. Figure S6: Representative image of the membranes used for the measurement of the apoptotic regulator proteins with the Human Apoptosis Array Kit. Each membrane was treated with a different derivative, as well as DMEM medium and DMSO for controls. Each protein is represented in duplicates on the membranes. The highly dense pairs in the corners of the membranes were used as internal controls. Table S1: Excerpt from the raw data of the cell cycle analysis PCR experiments done on MCF7 cells. PCR control wells (positive and no-template controls to verify assay performance), and gDNA wells (genomic DNA to check primer specificity and potential DNA carryover) were built in the used premade plates. The following quality control rules were explemented: no-template controls were required to be undetermined or to amplify only at very late cycles (Cq ≥ 38); earlier amplification indicated contamination, and the affected assay was excluded. gDNA wells were required to be undetermined or ≥10 cycles later. Wells underwent the ΔCt/ΔΔCt analysis and relative quantification was performed using the chosen reference gene (in this case the GAPDH).

## Abbreviations

The following abbreviations are used in this manuscript:

BCL2	B-cell lymphoma 2
Bort	Bortezomib
CCNA1	Cyclin A1
CCNA2	Cyclin A2
CCNB2	Cyclin B2
CCND1	Cyclin D1
CCND2	Cyclin D2
CCND3	Cyclin D3
CCNE1	Cyclin E1
CD36	Cluster of Differentiation 36
CDC25	Cell Division Cycle 25
CDC25A	Cell Division Cycle 25A
CDC25B	Cell Division Cycle 25B
CDC25C	Cell Division Cycle 25C
CDK1	Cyclin-dependent kinase 1
CDK2	Cyclin-dependent kinase 2
CDK4	Cyclin-dependent kinase 4
CDK6	Cyclin-dependent kinase 6
cDNA	Complementary DNA
CYP2E1	Cytochrome P450 2E1
CytC	Cytochrome c
DMSO	Dimethyl sulfoxide
E2F1	E2F Transcription Factor 1
E2F2	E2F Transcription Factor 2
E2F3	E2F Transcription Factor 3
E2F5	E2F Transcription Factor 5
EC80	Effective Concentration 80%
ER	Estrogen Receptor
FADD	Fas-Associated Death Domain
Fas/TNFRSF6/CD95	Fas cell surface death receptor
GADH	Glutamate Dehydrogenase
GPX1	Glutathione Peroxidase 1
HGPRT	Hypoxanthine-guanine phosphoribosyltransferase
HIF1a	Hypoxia-Inducible Factor 1-alpha
HO1/HMOX1/HSP32	Heme Oxygenase 1
HO2/HMOX2	Heme Oxygenase 2
HSP27	Heat Shock Protein 27
HSP60	Heat Shock Protein 60
HSP70	Heat Shock Protein 70
HSPD1	Heat Shock Protein Family D Member 1
IC50	Half Maximal Inhibitory Concentration
IL1B	Interleukin-1 Beta
INS	Insulin
LDHA	Lactate Dehydrogenase A
MPO	Myeloperoxidase
mRNA	Messenger RNA
NOS1	Nitric Oxide Synthase 1
NOX1	NADPH Oxidase 1
PARK7	Parkinsonism-associated Deglycase
PON2	Paraoxonase 2
PRDX1	Peroxiredoxin 1
PRDX3	Peroxiredoxin 3
PTGS2	Prostaglandin-Endoperoxide Synthase 2 (also known as COX-2)
RB1	Retinoblastoma Protein
RBL1	Retinoblastoma-like Protein 1
RBL2	Retinoblastoma-like Protein 2
ROS	Reactive Oxygen Species
ROS1	ROS Proto-Oncogene 1
SERM	Selective Estrogen Receptor Modulator
SERPINE1	Serpin Family E Member 1
SMAC/Diablo	Second Mitochondria-derived Activator of Caspases
SOD3	Superoxide Dismutase 3
TBP	TATA-Binding Protein
TFDP1	Transcription Factor Dp-1
TRAIL R2/DR5	TNF-Related Apoptosis-Inducing Ligand Receptor 2/Death Receptor 5
TRAILR1/DR4	TNF-Related Apoptosis-Inducing Ligand Receptor 1/Death Receptor 4
UCP1	Uncoupling Protein 1
