# Supplementary material for: Comparative Study of Ferrocene- and Indene-Based Tamoxifen Derivatives of Different Molecular Flexibility on High-Mortality Cancer Cell Lines
Source: Pharmaceuticals (Basel). 2025 Sep 20;18(9):1417. doi: 10.3390/ph18091417 (PMC12472596; doi:10.3390/ph18091417)
Supplement: Supplementary file 1 [file pharmaceuticals-18-01417-s001.zip › pharmaceuticals-3841522-supplementary.pdf]

Supplemetary Material

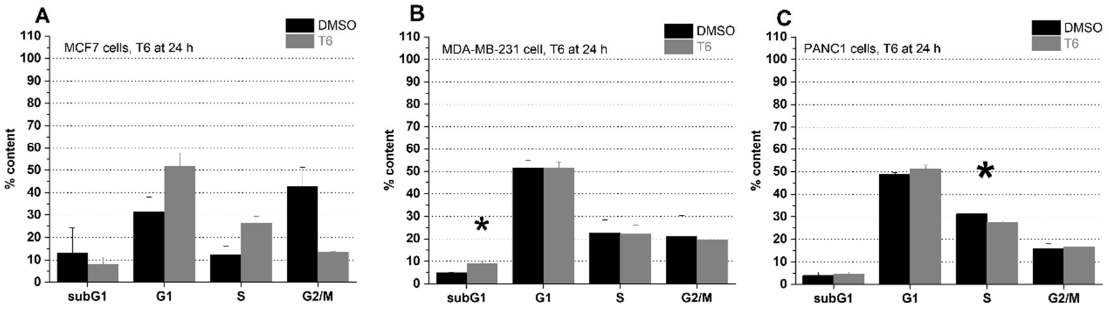

Figure S1: Impact of T6 on the cell cycle at 24 hours following treatment.

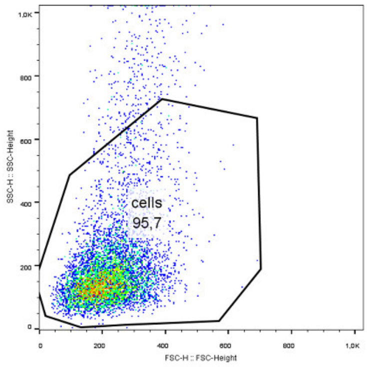

Example for gating
(MCF7 cells after T6 treatment)

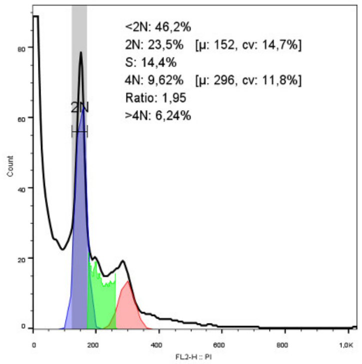

MCF7 cells 48 hours after T6 treatment

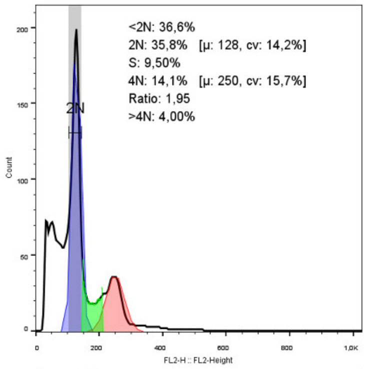

MDA-MB231 cells 48 hours after
T6 treatment

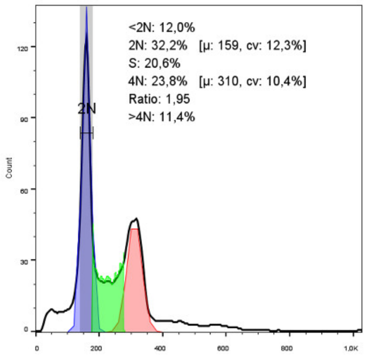

PANC1 cells 48 hours after T6 treatment

Figure S2: Flow cytometry output for the cell cycle analysis. Experiments were done in duplicates. For the sake of better representation, results from only one of the parallel samples are represented.

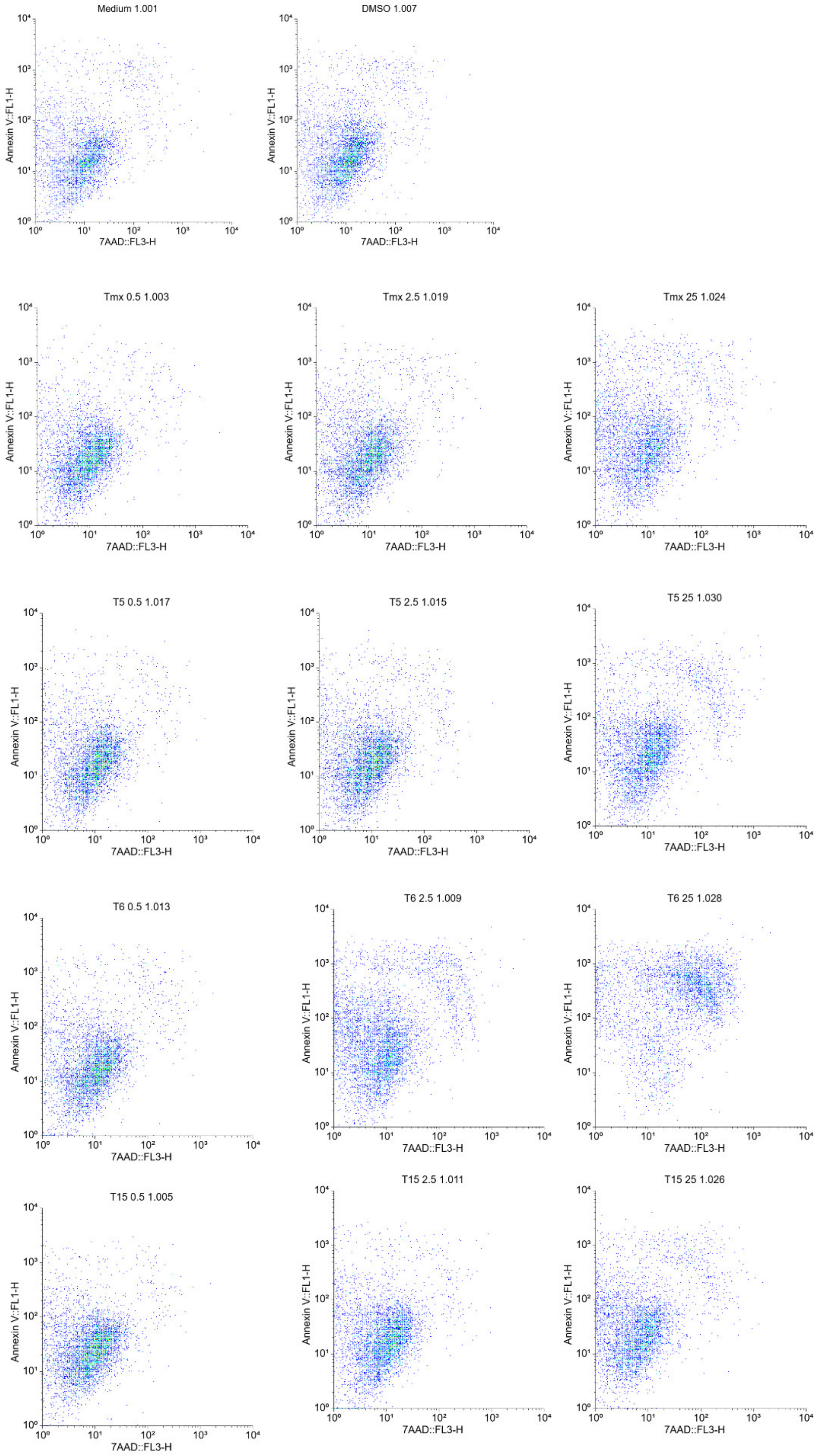

Figure S3: Flow cytometry output for the apoptosis measurements on MCF7 cells. Experiments were done in duplicates, 24 hours following treatment. For the sake of better representation, results from only one of the parallel samples are represented and where both the Annexin V and the 7AAD stains were used.

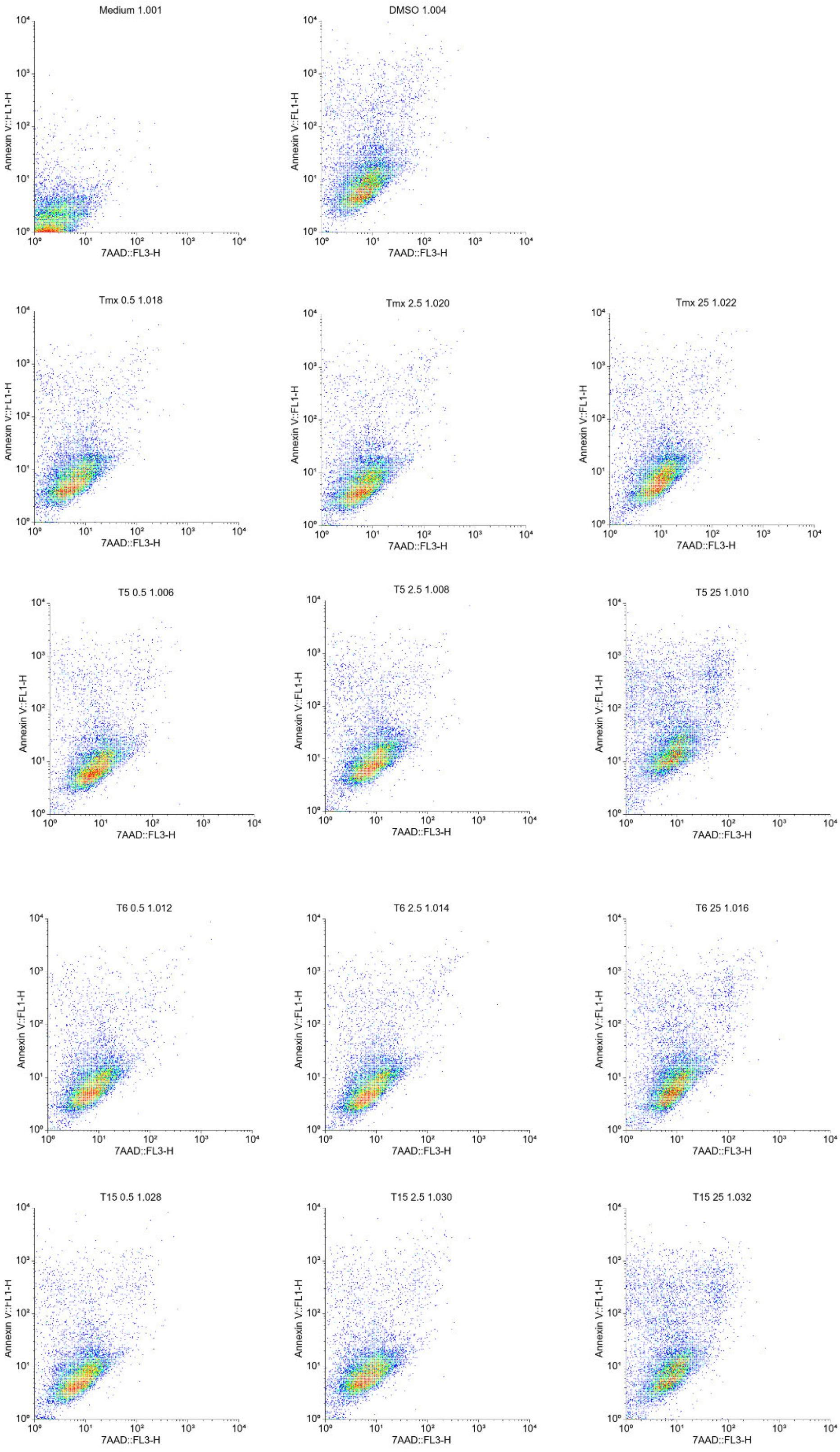

Figure S4: Flow cytometry output for the apoptosis measurements on MDA-MB231 cells. Experiments were done in duplicates, 24 hours following treatment. For the sake of better representation, results from only one of the parallel samples are represented and where both the Annexin V and the 7AAD stains were used.

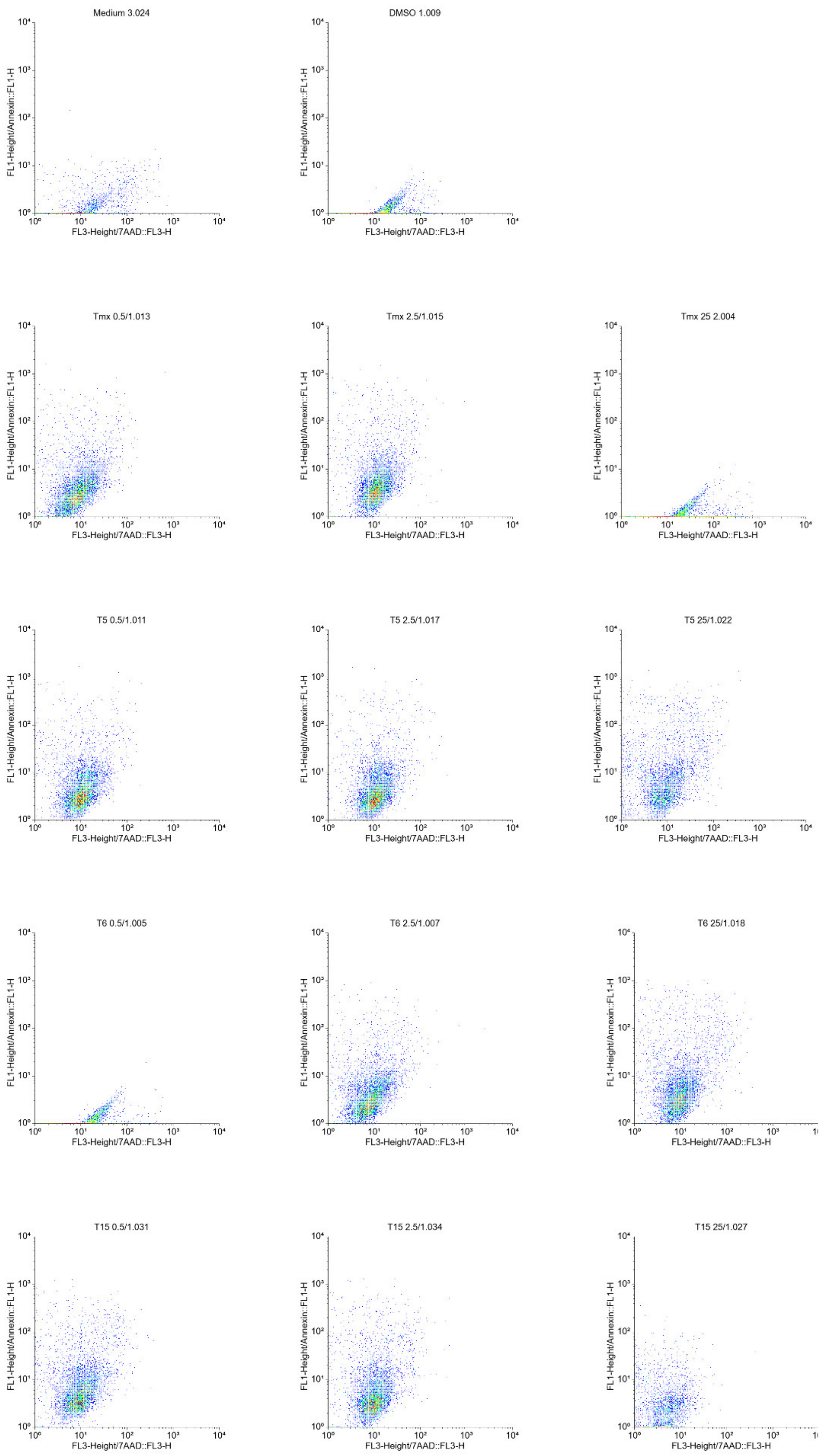

Figure S5: Flow cytometry output for the apoptosis measurements on PANC1 cells. Experiments were done in duplicates, 24 hours following treatment. For the sake of better representation, results from only one of the parallel samples are represented and where both the Annexin V and the 7AAD stains were used.

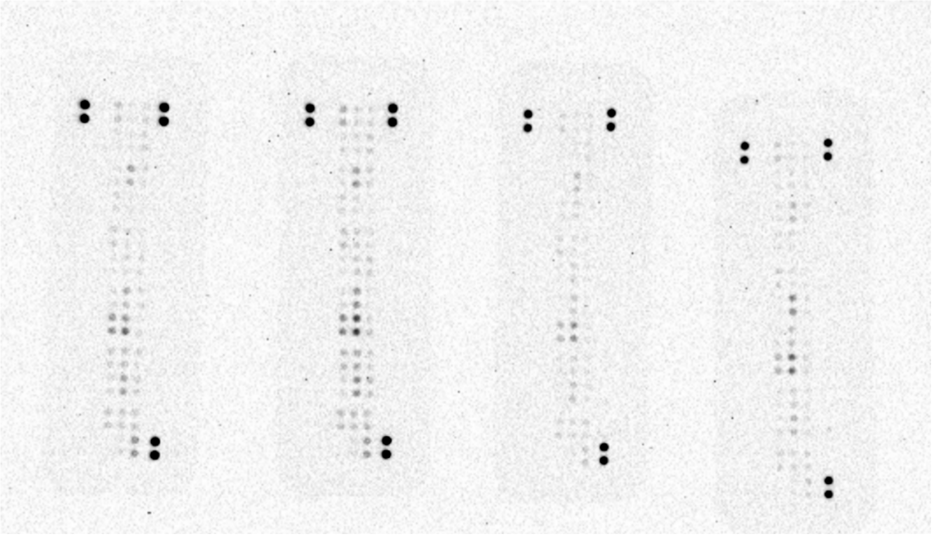

Figure S6: Representative image of the membranes used for the measurement of the apoptotic regulator proteins with the Human Apoptosis Array Kit. Each membrane was treated with a different derivative as well as DMEM medium and DMSO for controls. Each protein is represented in duplicates on the membranes. The highly dense pairs in the corners of the membranes were used as internal controls.

Table S1: Excerpt from the raw data of the cell cycle analysis PCR experiments done on MCF7 cells. PCR control wells (positive and no-template controls to verify assay performance), and gDNA wells (genomic DNA to check primer specificity and potential DNA carryover) were built in the used premade plates. The following quality control rules were explemented: no-template controls were required to be undetermined or to amplify only at very late cycles (Cq ≥ 38); earlier amplification indicated contamination, and the affected assay was excluded. gDNA wells were required to be undetermined or ≥10 cycles later. Wells underwent the ΔCt/ΔΔCt analysis and relative quantification was performed using the chosen reference gene (in this case the GAPDH).

Target	Sample	Cq			Mean Cq	dCq	ddCq	2^-ddCq
CDK4	DMSO	21,50511	20,96809	21,51231	21,3285	4,461554	-3,14462	8,843528
CDK6	DMSO	25,65745	24,57322	32,10074	27,4438	10,57685	2,970677	0,127567
E2F1	DMSO	25,29151	24,6417	26,09645	25,34322	8,476271	0,870094	0,547111
GAPDH	DMSO	16,85367	16,58279	17,16439	16,86695	0		
CDK4	T15		21,78266	21,53865	21,66066	-1,4731	-5,93465	61,16585
CDK6	T15	30,17343	26,26569	25,43615	27,29176	4,158001	-6,41885	85,55925
E2F1	T15		27,1752	26,89607	27,03564	3,901881	-4,57439	23,82476
GAPDH	T15	17,15942	18,18281	34,05904	23,13376	0		
CDK4	T5	20,17585	21,31255	20,40285	20,63042	1,045988	-3,41557	10,67058
CDK6	T5	24,1337	25,40945	26,21248	25,25187	5,667444	-4,90941	30,05242
E2F1	T5	23,62818	32,18994	25,28295	27,03369	7,449261	-1,02701	2,037796
GAPDH	T5	22,46471	18,24013	18,04845	19,58443	0		
CDK4	T6	23,99	25,58	28,44	26,00	5,46	1,00	0,500
CDK6	T6	30,40	37,87	28,53	32,26	11,72	1,15	0,452
E2F1	T6	28,91		29,06	28,98	8,44	-0,03	1,024
GAPDH	T6	20,54	20,54		20,54	0,00		
CDK4	Tamoxifen	25,40563	24,15861	25,97226	25,17884	4,198512	-3,40766	10,61229
CDK6	Tamoxifen	29,98389	28,05342		29,01865	8,038329	0,432152	0,741155
E2F1	Tamoxifen	30,89096	29,66391	30,05007	30,20165	9,221322	1,615145	0,326432
GAPDH	Tamoxifen	21,39611	20,78607	20,7588	20,98032	0		
